# The Risk of Hospitalizations with Injury Diagnoses in a Matched Cohort of Children and Adolescents with and without Attention Deficit/Hyperactivity Disorder in Germany: A Database Study

**DOI:** 10.3389/fped.2017.00220

**Published:** 2017-10-24

**Authors:** Christina Lindemann, Ingo Langner, Tobias Banaschewski, Edeltraut Garbe, Rafael T. Mikolajczyk

**Affiliations:** ^1^Department of Clinical Epidemiology, Leibniz Institute for Prevention Research and Epidemiology – BIPS, Bremen, Germany; ^2^Medical School Hannover, Hannover, Germany; ^3^Department of Child and Adolescent Psychiatry and Psychotherapy, Central Institute of Mental Health, Mannheim, Germany; ^4^Core Scientific Area ‘Health Sciences’ at the University of Bremen, Bremen, Germany; ^5^ESME – Epidemiological and Statistical Methods Research Group, Helmholtz Centre for Infection Research, Braunschweig, Germany; ^6^Institute for Medical Epidemiology, Biometrics and Computer Science, Martin-Luther-University Halle-Wittenberg, Halle (Saale), Germany

**Keywords:** attention deficit/hyperactivity disorder, injuries, subtype of injuries, sex differences, administrative data, claims data, Germany

## Abstract

**Background:**

Attention deficit/hyperactivity disorder (ADHD) is a common neuropsychiatric disorder in children and adolescents worldwide, and children with ADHD have elevated risk of injuries. Our aim was to assess the risk of hospitalizations with injury diagnoses and their various subtypes in children and adolescents with newly diagnosed ADHD compared to those without ADHD, as well as to study sex effects on this risk in the setting of the German health care system.

**Methods:**

The German Pharmacoepidemiological Research Database, in which 20 million insured from four statutory health insurances in Germany are included, was used to set up a matched cohort study of 3- to 17-year-old children and adolescents with and without ADHD. We calculated age-specific incidence rates and used Cox regression to obtain hazard ratios (HRs) for hospitalizations with injury diagnoses. We used the injury mortality diagnosis matrix for classification of injuries.

**Results:**

The matched cohort comprised a total of 75,300 children. The age-specific incidence rates for hospitalization with injury diagnosis for males with ADHD displayed a u-shaped form with highest incidences in the in the age groups 3–6 years [26.2 per 1,000 person-years; 95% confidence interval (CI) 20.5–33.0] and 18–21 years (28.6; 22.4–36.0). Girls with ADHD were less affected in younger age-groups, but the incidence rate for 18–21 year olds was similar to boys with ADHD (26.4; 17.4–38.4). The adjusted HR for children with ADHD was 1.40 (95% CI 1.30–1.49) compared to non-affected children. With respect to nature of injury, ADHD was associated with hospitalization with injury diagnoses of the internal organs, open wounds, and contusions but not with other injuries. With respect to body regions, children with ADHD were more prone to hospitalizations with injuries of the abdomen, traumatic brain injuries, other head injuries, and system-wide injuries such as poisoning and intoxication. No significant associations were seen for the other body regions. Differences between sexes were only seen for system-wide injuries.

**Conclusion:**

Children and adolescents with ADHD are at an increased risk for hospitalizations with diagnoses of injuries compared to non-affected children. Despite differences in health-care systems, the risk increase is at a similar level in Germany as in other countries.

## Introduction

Attention deficit/hyperactivity disorder (ADHD) is one of the most common psychiatric disorders in children worldwide. The core symptoms include inattention, hyperactivity, and impulsivity (1). The worldwide pooled prevalence of ADHD among children below 18 years is 5.3% (95% confidence interval (CI) 5.0–5.7) (2). In Germany, the prevalence estimates ranged from 2.1 to 4.8%, depending on how the diagnosis was assessed (3–5). From the public health point of view, ADHD has a substantial impact, with higher utilization of the health care system and higher mortality of persons with ADHD compared to those without ADHD, mainly due to injuries (6–9).

Studies using claims data to assess the association between injuries and ADHD have mostly been conducted in the US and Canada using populations with different case assessments, sample sizes, age-ranges, and in different years. In total, they showed an elevated risk up to a factor of five for those injuries which require hospital treatment among children and adolescents with ADHD compared to individuals without ADHD (10–16). In addition to assessing the overall higher risk of injuries, also the severity and the spectrum of injuries in children and adolescents with ADHD were investigated (11, 14, 15, 17, 18). Children and adolescents with ADHD suffer from more severe injuries and are more often in need of hospital treatment following injury than those without ADHD (13, 17). In previous studies, ADHD was associated specifically with an increased risk of traumatic brain injuries (TBI), poisoning and intoxication, burns and open wounds, as well as fractures compared to children without ADHD (18–23). Even though the majority of previous studies used large databases, not all of them had enough cases to analyze all relevant subtypes of injuries. Some researchers were only able to analyze a selection of injuries depending on the underlying database (22, 23).

The diagnostic methods used for ADHD vary between countries, but the difference can be even larger in their practical application in different health care systems, for example regarding the diagnostic threshold (24, 25). This influences the prevalence of diagnosed ADHD, and consequently, also impacts the incidence of injuries for children and adolescents with ADHD diagnosis. Therefore, the risk of injury among individuals with an ADHD diagnosis can differ by country, justifying the need of country specific analyses.

Sex differences in injury subtypes are known for children and especially for adolescents (26). Due to their risk-taking behavior, boys are in general more often seriously injured than girls (27), but during adolescence girls are more prone to experience injuries related to suicide and self-harm than boys (28–30). Since contradictory results for the sex-specific risks of children with ADHD were found (11, 14–16, 18–21), it remains unclear whether ADHD modifies these sex differences. Again, the results from different countries can be influenced by the local modalities in regard to diagnosis, treatment, and cultural differences in sex-related behavior of ADHD.

Taking advantage of a large cohort study of ADHD children and controls, addressing various research questions (5, 31–34), we aimed to: (a) determine the risk of hospitalizations with injury diagnoses (overall, by subtype of injury, and affected body regions) in the matched cohort of 3- to 17-year-old children and adolescents with and without ADHD as observed in German health care setting, and (b) to assess how the effects of ADHD on the risk of hospitalization with injury diagnoses depend on sex.

## Materials and Methods

### Data Source

The German Pharmacoepidemiological Research Database (GePaRD), which comprises data of 20 million insured (one fourth of the population) from all over Germany, was used to select a matched cohort with children with ADHD diagnoses. The database is described elsewhere (35, 36). In brief, GePaRD consists of claims data from four statutory health insurances (SHIs) covering all geographical areas of Germany. For each included insurant, demographic data such as age, sex, and residence is available as well as information about in- and outpatient treatment, including ambulatory physicians’ visits, ambulatory prescriptions, and hospital admissions. In- and outpatient diagnoses are coded according to the German modification of the International Classification of Diseases version 10 (ICD-10-GM), in- and outpatient procedures are coded according to the Operations and Procedures Coding System (OPS) and outpatient procedures according to the “einheitlicher Bewertungsmaßstab.”

### Ethics and Legal Regulations

According to German Social Insurance Code (§75, volume 10), no informed consent was needed from the insurants for this study. The use of the data for this study was approved by all four SHIs, the Federal Ministry of Health, and the Senate of the Federal State of Bremen. Since this study was based on routinely collected pseudonymized data, no ethical approval was needed. Within GePaRD exists a data protection concept, which protects the privacy rights of each insurant.

### Study Population

#### Study Population and Identification of ADHD Cases

Study entry for the children and adolescents was between 2005 and 2007. Eligible subjects had to reach an age between 3 and 17 years in their year of study entry, had to have a continuous insurance period of 1 year free of ADHD indicators like diagnoses or prescriptions for MPH or atomoxetine (ATX) directly before study entry, and had to have a continuous insurance period of at least one year subsequent to their study entry. Study participants were followed until the 31st December 2009 or their end of insurance, whichever came first. Among these, newly diagnosed ADHD-cases were identified by the following algorithm based on ICD-10-GM diagnoses and drug dispensations: children had to have received either one inpatient diagnosis F90.0 or F90.1, or at least two outpatient diagnoses F90.0, F90.1, or F90.9 (of which one had to be F90.0 or F90.1) within a period of 1 year, or a dispensation of methylphenidate [MPH, Anatomical Therapeutic Chemical (ATC) code: N06BA04] or ATX (ATC code: N06BA09) together with one outpatient diagnosis F90.0 or F90.1 within 1 year. The first date of the events considered for the case identification as the index date of the case.

### Study Design

This cohort study used a matched design. We included identified ADHD-cases with an index date situated between 2005 and 2007. For each of them, a subject without ADHD was matched on sex, birth year, region of residence and insurance company and the potential follow up of the matched subject had to be at least as long as of the ADHD case. Study entry date for both of a matched pair was the index date of the ADHD case and consequently both had to fulfill the defined inclusion criteria at this date. Further, the follow-up of the matched subject was censored at the follow-up end of the corresponding ADHD case. Subjects with an ADHD case index date after 2007 were excluded before matching.

### Ascertainment of Injuries

For the ascertainment of injuries, we used the injury mortality diagnosis (IMD) matrix developed by Fingerhut and Warner (37) to standardize the presentation and assessment of ICD-10 “S” and “T” codes for injuries. The IMD matrix is an enhancement of the Barell Matrix which was designed to classify injuries based on ICD-9 CM codes (38). The IMD matrix has two axes: one concerning the body region and one concerning the nature of the injury. It holds 43 “body regions” and 20 “nature of injury” categories and can be collapsed into fewer categories to present a more comprehensive and interpretable picture. Injuries affecting the entire body such as poisoning or intoxication are also included in the IMD matrix and classified as system-wide injuries. We modified the IMD matrix according to the German Modification of the ICD-10 and included for example some T00 codes which were not considered in the US ICD version and therefore not used in the original IMD matrix (see Table S1 in Supplementary Material).

### Statistical Analysis

For the descriptive analyses, the study population was stratified by sex, 3-year age classes (3 to <6, 6 to <9, 9 to <12, 12 to <15, 15 to <18, 18+), case status (ADHD yes/no), and nature of the injury or injured body region (5). Based on the study population of newly diagnosed ADHD cases and their matched subjects, incidence rates for hospitalizations (with overnight stay) due to any injury were calculated as cumulative number of events divided by cumulative person-time. The corresponding 95% CI were calculated following the method recommended by Newcombe and Altman (39). Subsequently, we estimated the proportions of children with at least one hospitalization with a specific subtype of injury diagnosis during 36 months of follow-up using Kaplan-Meier-estimator in PROC LIFETEST in SAS with the corresponding 95% CI. To assess the influence of ADHD on the risk of hospitalization with diagnoses of injuries, Cox regression analyses were conducted for injuries overall and subtypes of injuries for which more than 150 cases were ascertained in the study population. Cohort members were censored at the time of an injury or the end of insurance, whichever came first. The regression models were adjusted for age at hospitalization and sex. In addition, we investigated if the risk of injuries associated with ADHD depended on sex by assessing the interaction between sex and ADHD status.

## Results

**Figure 1:**
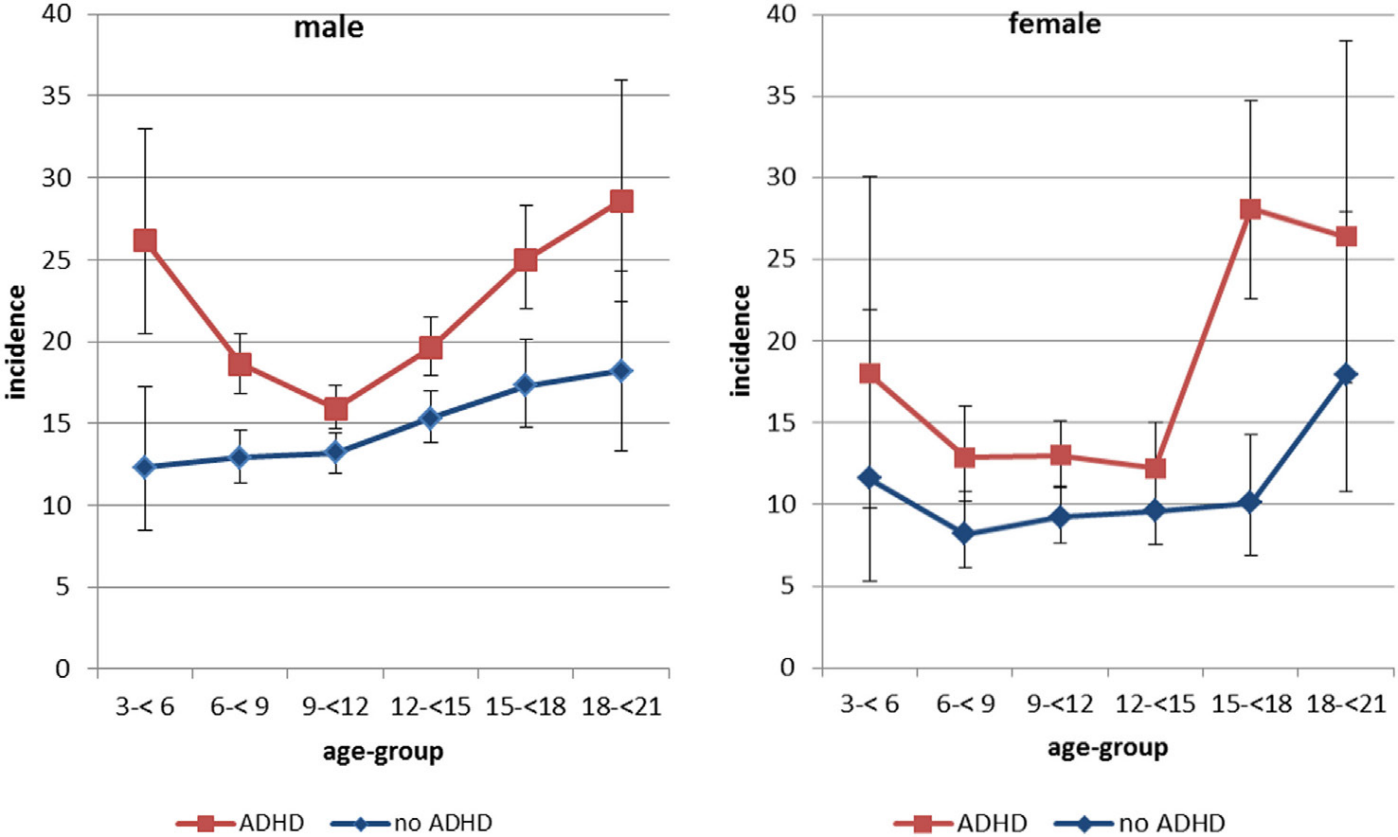
Incidence of hospitalizations with at least one injury diagnosis by ADHD status, age-group, and sex*. *Per 1,000 person-years. Note: Whiskers represent 95% confidence intervals.

In the years 2005–2007, 37,650 children and adolescents 3–17 years old with a new ADHD diagnosis were identified and matched 1:1 to subjects without ADHD. Follow-up of the cohort until 2009 accumulated to a total of 254,985 person-years. During this time, we identified 3,995 inpatient treatments due to injuries. The age-specific incidence for hospitalizations with at least one injury diagnosis showed an almost u-shaped form among boys with ADHD, with highest incidences in the age-groups 3–6 and 18–21 years (Figure 1). Among boys without ADHD, there was a slight increase of risk with age, but no indication of a u-shape. The incidence of injuries for girls was lower at younger ages, but strongly increased in the oldest age-groups studied and reached levels observed in boys.

Boys with or without ADHD were most often hospitalized with diagnoses of injuries of the internal organs, fractures, and contusions (classification according to nature of injuries, Table 1).

**Table 1:** Estimated % experiencing hospitalizations with injury diagnoses during follow-up of 36 months, by nature of injury.^a^

	% experiencing hospitalizations with injury diagnoses during individual follow-up (95% confidence interval)			
	Boys		Girls	
Nature of injury	ADHD	No ADHD	ADHD	No ADHD
Fracture	1.73 (1.58–1.90)	1.37 (1.24–1.53)	1.11 (0.91–1.36)	1.00 (0.80–1.24)
Dislocation	0.09 (0.06–0.14)	0.10 (0.07–0.15)	0.09 (0.04–0.18)	0.08 (0.04–0.18)
Internal organ injury	1.77 (1.62–1.94)	1.29 (1.16–1.44)	1.32 (1.10–1.59)	0.88 (0.70–1.10)
Open wound	1.23 (1.10–1.37)	0.85 (0.75–0.97)	0.71 (0.55–0.91)	0.46 (0.33–0.63)
Amputation	0.01 (0.00–0.03)	0.01 (0.00–0.03)	0.01 (0.00–0.08)	0 (.–.)*
Injuries to blood vessels	0.03 (0.02–0.07)	0.02 (0.01–0.05)	0.01 (0.00–0.08)	0.01 (0.00–0.08)
Superficial and contusion	1.39 (1.25–1.54)	0.93 (0.82–1.05)	1.07 (0.87–1.31)	0.74 (0.57–0.95)
Crushing	0.01 (0.00–0.03)	0 (.–.)*	0 (.–.)*	0 (.–.)*
Burn	0.08 (0.05–0.12)	0.04 (0.02–0.08)	0.08 (0.04–0.18)	0.05 (0.02–0.13)
Effect of foreign bodies entering orifice	0.10 (0.07–0.15)	0.03 (0.02–0.07)	0.11 (0.06–0.20)	0.01 (0.00–0.09)
Other effects of external causes	0.10 (0.07–0.15)	0.09 (0.06–0.13)	0.13 (0.07–0.24)	0.01 (0.00–0.08)
Poisoning	0.12 (0.09–0.17)	0.03 (0.02–0.07)	0.32 (0.22–0.47)	0.03 (0.01–0.10)
Toxic effects	0.25 (0.20–0.32)	0.13 (0.09–0.18)	0.28 (0.19–0.42)	0.08 (0.04–0.16)
Multiple injuries	0.03 (0.01–0.06)	0.02 (0.01–0.05)	0.02 (0.01–0.09)	0.02 (0.01–0.09)
Other specified injury	0.46 (0.38–0.55)	0.37 (0.30–0.45)	0.25 (0.16–0.38)	0.19 (0.12–0.32)
Unspecified injury	0.33 (0.27–0.41)	0.23 (0.18–0.30)	0.26 (0.17–0.40)	0.16 (0.10–0.28)

*^a^Classified according to IMD matrix*.

*For injuries which occurred in 0% no confidence intervals were estimated.

The same types of injuries were most common among girls, but the proportion of girls experiencing hospitalizations with those injuries was lower than for boys. For these diagnoses, the risk was higher among individuals with ADHD than without, while for several types of injuries there was no difference associated with the ADHD status. Hospitalizations with diagnosis of poisoning were particularly high among girls with ADHD. With respect to the body region, most of the hospitalizations occurred with diagnoses of TBI and injuries of the upper extremity (Table 2). For most body regions, a higher proportion of boys than of girls experienced hospitalizations, respectively. Only for some body regions, the proportion was higher among individuals with ADHD than without ADHD.

**Table 2:** Estimated % experiencing hospitalizations with injury diagnoses during follow-up of 36 months, by body region.^a^

	% experiencing hospitalizations with injury diagnoses during individual follow-up (95% confidence interval)			
	Boys		Girls	
Affected body region	ADHD	No ADHD	ADHD	No ADHD
Traumatic brain injury (TBI)	1.87 (1.71–2.04)	1.32 (1.19–1.47)	1.38 (1.15–1.65)	0.91 (0.73–1.13)
Other head	0.85 (0.74–0.97)	0.59 (0.50–0.69)	0.66 (0.51–0.86)	0.36 (0.25–0.51)
Neck	0.02 (0.01–0.05)	0.02 (0.01–0.04)	0.01 (0.00–0.08)	0 (.–.)*
Head and neck. other	0.03 (0.02–0.07)	0 (0.00–0.03)	0 (.–.)*	0.01 (0.00–0.09)
Spinal cord	0 (.–.)*	0.01 (0.00–0.04)	0.01 (0.00–0.08)	0.01 (0.00–0.08)
Vertebral column	0.18 (0.13–0.24)	0.14 (0.10–0.20)	0.20 (0.13–0.33)	0.13 (0.07–0.24)
Thorax	0.21 (0.16–0.27)	0.15 (0.11–0.20)	0.17 (0.10–0.28)	0.20 (0.12–0.33)
Abdomen	0.21 (0.16–0.27)	0.10 (0.07–0.15)	0.12 (0.07–0.22)	0.11 (0.06–0.21)
Pelvis and lower back	0.16 (0.11–0.21)	0.09 (0.06–0.14)	0.28 (0.19–0.42)	0.14 (0.08–0.25)
Abdomen, lower back, and pelvis	0.34 (0.28–0.42)	0.21 (0.16–0.27)	0.29 (0.19–0.43)	0.14 (0.08–0.25)
Other trunk	0.12 (0.08–0.16)	0.06 (0.04–0.10)	0.15 (0.09–0.25)	0.02 (0.01–0.09)
Upper extremity	1.65 (1.50–1.82)	1.22 (1.09–1.36)	1.02 (0.83–1.26)	0.82 (0.65–1.04)
Hip	0.04 (0.02–0.07)	0.02 (0.01–0.05)	0.03 (0.01–0.10)	0.02 (0.01–0.09)
Other lower extremity	0.81 (0.71–0.93)	0.61 (0.52–0.71)	0.59 (0.45–0.77)	0.45 (0.33–0.63)
Multiple body regions	0.06 (0.04–0.10)	0.05 (0.03–0.09)	0 (.–.)*	0.02 (0.00–0.13)
System-wide injuries^b^	0.50 (0.42–0.60)	0.26 (0.20–0.33)	0.70 (0.54–0.90)	0.12 (0.07–0.22)
Unspecified	0.19 (0.15–0.25)	0.15 (0.11–0.20)	0.11 (0.05–0.20)	0.07 (0.03–0.17)

*^a^Classified according to IMD matrix*.

*^b^e.g., poisoning, intoxication*.

*For injuries which occurred in 0% no confidence intervals were estimated.

**Figure 2:**
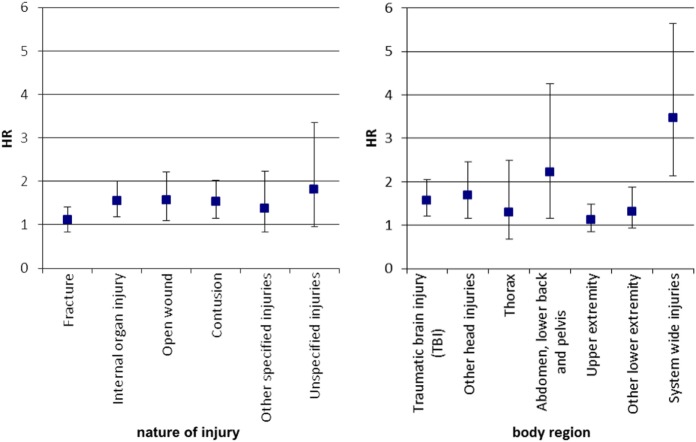
Adjusted hazard ratio of hospitalization with injury diagnoses for children with ADHD versus those without*. *Adjusted for age at hospitalization, and sex; only injury categories with at least 150 events were analyzed. Note: cox regression analysis, HR, hazard ratio, Whiskers indicate 95% confidence intervals.

The overall adjusted hazard ratio (HR) for a hospitalization with injury diagnosis for children and adolescents with ADHD was 1.40 (95% CI 1.30–1.49) compared to those without ADHD. The results were similar for hospitalizations with injuries of internal organs, open wounds, and contusions, while other categories of injuries like fractures, other specified injuries (see IMD matrix), and unspecified injuries were not associated with an elevated risk in the presence of ADHD (Figure 2). Concerning the affected body region, children with ADHD had a higher risk for hospitalizations with injuries of the abdomen, TBI, other head injuries, and system-wide injuries than children without ADHD. While most of these HRs were between 1 and 1.5, the HR of hospitalization due to system-wide injuries was substantially higher (HR 3.47; 95% CI 2.14–5.64).

**Figure 3:**
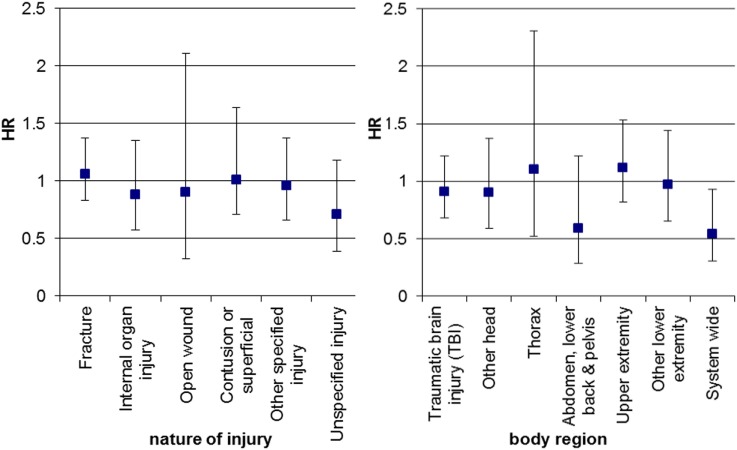
Sex-related difference on the influence of ADHD on hospitalization with injuries for boys versus girls*. Cox regression analysis, HR, hazard ratio for the interaction of sex and ADHD status for boys versus girls adjusted for age at hospitalization and the main effects of sex and ADHD case status, whiskers indicate 95% confidence intervals. *Only injury categories with at least 150 events were included.

The risk of hospitalizations with injury diagnoses among individuals with ADHD and without ADHD was not modified by sex, with the exception of system-wide injuries where the ADHD associated risk increase was less pronounced in boys compared to girls (Figure 3).

## Discussion

Taking advantage of a large matched cohort of children and adolescents with and without ADHD in German health insurance data, we studied the relative risk of hospitalizations with injury diagnoses associated with ADHD and how it depends on sex. We confirmed the higher risk of hospitalizations with injury diagnoses among children with newly diagnosed ADHD compared to sex- and age-matched controls without ADHD. We observed significantly elevated HRs for injuries of the abdomen, traumatic brain injury as well as for system-wide injuries among children with ADHD. Children or adolescents with ADHD also had a higher risk for hospitalizations with diagnoses of injuries of the internal organs, open wounds, and contusions compared to those non-affected. We showed no differences by sex with respect to ADHD for all studied subtypes of injuries apart from the hospitalizations with system-wide injuries where the risk increase related to ADHD was less pronounced in boys compared to girls.

These findings are consistent with previous studies, in which children and adolescents with ADHD displayed a higher risk of injuries than healthy controls (10, 12, 14, 21), and also in line with previous studies assessing injury patterns among children with ADHD (11, 21, 23, 40). Although the country-specific setting might influence if children are diagnosed with ADHD, depending on diagnostic modalities and a threshold of symptom severity applied in routine health care (for example reflected in regional differences in prevalence or sex ratio of diagnosed cases) (41, 42), and consequently in those diagnosed, the risk of injuries can be different, this does not seem to translate into different findings in our analysis.

The risk estimates of various injury patterns were elevated by a factor of two, except for system-wide injuries for which the HR was 3.5, indicating a particularly strongly increased risk. Among studies reporting the risk of poisoning, which is after intoxication the main type of system-wide injuries in children with ADHD, we only identified two reporting significantly elevated risk in children with ADHD compared to their non-affected controls (21, 43). The study by Rowe et al. (21) found a relative risk estimate of 2.3 (95% CI 1.1–4.8), which was similar to the risk estimates for other studied injury patterns in their study. The second study reported a relative risk estimate of 4.65 (95% CI 2.41–8.94) (43), which is even somewhat higher than our estimate of 3.47. Four other studies did not find a higher risk for children with ADHD of poisoning compared to children without ADHD (11, 12, 19, 23). Given the various settings of those studies, further research is necessary to explain these differences.

We also found a higher risk of system-wide injuries for girls with ADHD compared to boys with ADHD. This effect has not been reported in the past. Our findings are corroborated by the well-known fact that female adolescents are at a higher risk of self-related harm and intoxication than male adolescents (28–30). In line with these results, we observed the highest incidence rates for injuries in girls with ADHD in the age-groups 15–18 and 18–21 years.

The risk of hospitalization with injury diagnoses was generally higher for boys than for girls. This is consistent with studies in the general population (27). Potential reasons are differences in motor function, risk taking behavior, and misleading perception of dangerous situations. On the contrary, the sex influence on the risk associated with ADHD is less clear. Previous studies have reported contradictory results. Some studies found that boys with ADHD are at a higher risk of injuries than girls with ADHD (11, 16, 18–21), one study found comparable risks of boys and girls with ADHD (14), and another study found higher risks of girls with ADHD compared to boys with ADHD (15). Our results suggest that age of the participants can be to some degree responsible for these differences.

### Strengths and Limitations

The main strength of this study is the use of a large administrative database, which in several previous studies showed agreement with data representative for Germany (44, 45). The analysis of the injuries was based on the IMD Matrix, which allows a systematic assessment of the different injury categories.

However, the current study has also a number of limitations. As it is true for every study conducted with administrative data, information gathered in the database was not collected for research purposes. For example, the diagnostic criteria for ADHD were not standardized for the purpose of this study, but followed the routine diagnostic standard in the German health care system. In this study both, cases and controls, were assessed in the same database, and therefore affected on equal terms. Still, patients are more likely to be consistently recorded as ADHD cases in claims data if they have a more severe form of ADHD and/or receive treatment. Consequently, if preferentially persons with more severe forms of ADHD are included in our case group, this could inflate our estimates. On the other side, we did not account for any treatment of ADHD. Recent studies using case only designs indicate a risk reduction due to medication, particularly with respect to injuries being more specific for ADHD (32, 46, 47). In this sense, our estimates are potentially confounded by the fraction receiving medication and given effective treatment the underlying risk would be higher. Our case algorithm excluded children who were not continuously insured for 1 year after study entry, and thus those children who died possibly in consequence of injuries. This, however, likely had only a marginal impact on the risk estimates, since death due to injuries is a rare event in this age group. Also, our case algorithm included a one year ADHD-free lead time to define newly diagnosed ADHD-cases. In fact, some of these cases might have been diagnosed in the past. For the rare case that ADHD diagnosed in the older age-groups, this could have resulted in including also prevalent ADHD-cases.

We focused in our analysis on hospitalizations with injury diagnoses. The way the data is recorded does not allow determining beyond doubt that the injury was the cause for the hospitalization, but it is likely the case. Unfortunately, we were not able to study the injuries treated in outpatient and emergency room settings, because diagnoses are recorded only quarterly wise and can be continued if related visits take place. For example after hospitalization, injury diagnoses can be recorded in outpatient follow-up. In consequence, we were not able to establish injury events for the outpatient setting.

Finally, we were not able to address influence of socioeconomic status since our data does not provide this information. As ADHD is associated with a low socioeconomic status (3) and a low socioeconomic status is also a well-documented stand-alone risk factor for injuries (48), this might have resulted in an overestimation of the association between ADHD and injury risk in our study.

## Conclusion

In summary, our results indicate that the risk of children and adolescents with ADHD for hospitalizations with injury diagnoses was substantially higher compared to their non-affected controls. We conducted an extensive analysis for various injury patterns concerning the affected body region and the nature of injury. In this context, we found that besides of system-wide injuries, there were no differences by sex. Parents and teachers of children with ADHD need to be advised of the higher risk of injuries. Moreover, attention should be paid to specific risks for girls with ADHD during adolescence.

## Ethics Statement

The study is based on routine data and therefore exempt from ethical review.

## Author Contributions

CL, IL, TB, RM, and EG were involved in the acquisition and the design of the study. CL, IL, RM were involved in the statistical analyses. All authors gave substantial contribution to the interpretation of the data. All authors were involved in drafting the work or revising it critically for important intellectual content. All authors gave approval for the publication of this version.

## Conflict of Interest Statement

IL is working for a department that occasionally performs studies for pharmaceutical industries. EG, RM, and CL were at the time of study conduct employees of this department. The pharmaceutical companies include Bayer, Celgene, GlaxoSmithKline, Mundipharma, Novartis, Sanofi-Aventis, Sanofi Pasteur MSD, and STADA. EG has been a consultant to Bayer, Takeda, Nycomed, GlaxoSmithKline, Schwabe, Teva, Astellas, Consulting AstraZeneca, and Novartis. These activities are unrelated to the current topic of this study. TB served in an advisory or consultancy role for Actelion, Hexal Pharma, Lilly, Medice, Novartis, Oxford outcomes, Otsuka, PCM scientific, Shire, and Viforpharma. He received conference support or speaker’s fee by Medice, Novartis and Shire. He is/has been involved in clinical trials conducted by Shire & Viforpharma. He received royalities from Hogrefe, Kohlhammer, CIP Medien, Oxford University Press. The present work is unrelated to the above grants and relationships.

## Abbreviations

ADHD, attention deficit/hyperactivity disorder; CI, confidence interval; TBI, traumatic brain injury; GePaRD, German Pharmacoepidemiological Research Database; SHI, statutory health insurance; ICD-10-GM, International Classification of Diseases version 10; OPS, Operations and Procedures Coding System; EBM, einheitlicher Bewertungsmaßstab; MPH, methylphenidate; ATX, atomoxetine; IMD matrix, the injury mortality diagnosis matrix; HR, hazard ratio.
