# Supplementary material for: The Risk of Hospitalizations with Injury Diagnoses in a Matched Cohort of Children and Adolescents with and without Attention Deficit/Hyperactivity Disorder in Germany: A Database Study
Source: Front Pediatr. 2017 Oct 24;5:220. doi: 10.3389/fped.2017.00220 (PMC5660679; doi:10.3389/fped.2017.00220)
Supplement: Supplementary file 1 [file table_1.docx]

Table 1 Injury Mortality Diagnosis (IMD) Matrix (ICD-10)

|  |  | **Nature of injury** | | | | | | | | | | | | | | | |
| --- | --- | --- | --- | --- | --- | --- | --- | --- | --- | --- | --- | --- | --- | --- | --- | --- | --- |
| **Body region of injury** | | **Frac-ture** | **Dislo-cation** | **Inter-nal organ injury** | **Open wound** | **Amputa-tion** | **Blood vessel** | **Superficial and contusion** | **Crushing** | **Burn** | **Effect of foreign bodies entering orifice** | **Other effects of external causes** | **Poisoning** | **Toxic effects** | **Multiple injuries** | **Other specified injury** | **Unspecified injury** |
| **Head and Neck** | **Traumatic brain injury (TBI)** | S02(.0–.1, .3, .7–.9), T90.2 | **...** | S06, T90.5 | S01, T90.1 | **…** | **…** | **...** | S07 | **...** | **...** | **...** | **...** | **...** | S09.7 | S04.0, S09.8, T90(.4, .8) | S09.9, T90.9 |
|  | **Other head** | S02(.2, .4–.6) | S03(.0–.3) |  | S05(.2–.7), S08.0, S09.2 | S08(.1–.9) | S09.0 | S00, S05(.0–.1), T90.0 | **...** | T26 | T15–T16, T17(.0–.1), T18.0 | T33.0, T34.0 | **...** | **...** | **...** | S03(.4–.5), S04(.1–.9), S05.8, S09.1, T90.3 | S05.9 |
|  | **Neck** | S12(.8–.9) | S13(.2–.3) | **...** | S11 | S18 | S15(.0, .2–.9) | S10 | S17 | T27.0, T27.4 | T17(.2–.4) | T33.1, T34.1 | **...** | **...** | S19.7 | S13(.5–.6), S14(.3–.6), S16, S19.8 | S19.9 |
|  | **Head and neck, other** | **...** | **...** | **...** | **...** | **...** | **...** | **...** | **...** | T20, T28.0, T28.5, T95.0 | **...** | T35.2 | **...** | **...** | **...** | **...** | **...** |
| **Spine and upper back** | **Spinal cord** | **...** | **...** | S14(.0–.1), S24(.0–.1), S34(.0–.1, .3), T09.3, T91.3 | **...** | **...** | **...** | **...** | **...** | **...** | **...** | **...** | **...** | **...** | **...** | **...** | **...** |
|  | **Vertebral column** | S12(.0–.7), S22(.0–.1), S32(.0–.2), T08, T91.1 | S13(.0–.1), S23(.0–.1), S33(.0–.2) | S14.2 | **...** | **...** | S15.1 | **...** | **...** | **...** | **...** | **...** | **...** | **...** | **...** | S13.4, S23.3, S24.2, S33(.5–.7), S34(.2, .4), T09.4 | **...** |
| **Torso** | **Thorax** | S22(.2–.9) | S23.2 | S26.0, S27(.0–.6, .8–.9), T91.4 | S21 | S28.1 | S25 | S20 | S28.0 | T28(.1, .6) | T17.5 | T33.2, T34.2 | **...** | **...** | S27.7, S29.7 | S23(.4–.5), S24(.3–.6), S26.8, S29(.0, .8) | S26.9, S29.9 |
|  | **Abdomen** | **...** | **...** | S36 | S31(.1,.8) | **...** | S35(.0–.4) | S30.1 | **...** | **...** | T18(.2–.4) | **...** | **...** | **...** | **...** | **...** | **...** |
|  | **Pelvis and lower back** | S32(.3–.8) | S33(.3–.4) | S37 | S31(.0, .2–.5) | S38.2 | S35.5 | S30(.0, .2) | S38.0 | T28(.3, .8) | T18.5, T19 | **...** | **...** | **...** | **...** | S34.5 | **...** |
|  | **Abdomen, lower back & pelvis** | T02.1 | **...** | S39(.6–.7), T06.5, T91.5 | S31.7 | S38.3 | S35(.7–.9) | S30(.7–.9) | S38.1 | **...** | **...** | T33.3, T34.3, T35.3 | **...** | **...** | T03.1 | S34(.6, .8), S39(.0, .8) | S39.9 |
|  | **Other trunk** | T91.2 | **...** | **...** | T09.1 | T09.6 | **...** | T09.0 | T04.1 | T21, T27(.2–.3, .6–.7), T28(.2, .7), T95.1 | T17(.8–.9), T18(.1, .8–.9) | **...** | **...** | **...** | T09.2 | T09(.5, .8) | T09.9 |
| **Extremities** | **Upper extremity** | S42, S52, S62, T02(.2, .4), T10, T92(.1–.2) | S43(.0–.3), S53(.0–.1), S63(.0–.2) | **...** | S41, S51, S61, T01.2, T11.1, T92.0 | S48, S58, S68 T05(.0, .2), T11.6 | S45, S55, S65, T11.4 | S40, S50, S60, T00.2, T11.0 | S47, S57, S67, T04.2 | T22–T23, T95.2 | **...** | T33(.4–.5), T34(.4–.5), T35.4 | **...** | **...** | S49.7, S59.7, S69.7, T03.2, T11.2, T92(.3, .6) | S43(.4–.7), S44, S46, S49.8, S53(.2–.4), S54, S56, S59.8, S63(.3–.7), S64, S66, S69.8, T11(.3, .5, .8), T92(.4–.5, .8) | S49.9, S59.9, S69.9, T11.9, T92.9 |
|  | **Hip** | S72(.0–.2) | S73.0 | **...** | S71.0 | S78.0 | **...** | S70.0 | S77.0 | **…** | **...** | **...** | **...** | **...** | **...** | S73.1, S76.0 | **...** |
|  | **Other lower extremity** | S72(.3–.9), S82, S92, T02(.3, .5), T12, T93(.1–.2) | S83(.0–.1), S93(.0–.1, .3) | **...** | S71(.1–.8), S81, S91, T01.3, T13.1, T93.0 | S78(.1–.9), S88, S98, T05(.3, .5), T13.6 | S75, S85, S95, T13.4 | S70(.1–.9), S80, S90, T00.3, T13.0 | S77(.1–.2), S87, S97, T04.3 | T24–T25, T95.3 | **...** | T33(.6–.8), T34(.6–.8), T35.5 | **...** | **...** | S79.7, S89.7, S99.7, T03.3, T13.2, T93(.3, .6) | S74, S76(.1–.7), S79.8, S83(.2–.6), S84, S86, S89.8, S93(.2, .4–.6), S94, S96, S99.8, T13(.3, .5, .8), T93(.4–.5, .8) | S79.9, S83.7, S89.9, S99.9, T13.9, T93.9 |
| **Unclassifiable by body region** | **Multiple body regions** | T02(.8–.9) | **...** | **...** | T01.9 | T05(.8–.9) | T06.3 | T00(.8–.9) | T04(.8–.9) | T27.1, T27.5**,** T28.9 | **...** | T35(.0–.1, .6) | **...** | **...** | T03(.8–.9), T91.0 | T06(.2, .4), T91.8 | T07, T91.9, T94.0 |
|  | **System wide** | **...** | **...** | **...** | **...** | **...** | **...** | **...** | **...** | **...** | **...** | T66–T75 | T36–T50, T96 | T51–T65, T97 | **...** | T79(.0–.9), T98.2 | **...** |
| **Unspecified** | | T14.2 | **...** | **...** | T14.1 | **...** | T14.5 | T14.0 | **...** | T28.4, T30–T32, T95(.4, .8–.9) | T98.0 | T33.9, T34.9, T35.7 | **...** | **...** | T14.3, T14.7 | T14(.4, .6) | T14(.8–.9), T94.1, T98.1 |
